# The attractiveness of salient distractors to reaching movements is task dependent

**DOI:** 10.3758/s13414-020-01984-6

**Published:** 2020-02-05

**Authors:** Tom Nissens, Katja Fiehler

**Affiliations:** Experimental Psychology, Justus Liebig University Giessen, Otto-Behaghel-Strasse 10F, 35394 Giessen, Germany

**Keywords:** Reaching, Attention, Movement curvature, Physical salience, Visual search, Suppression

## Abstract

Previous studies in visual attention and oculomotor research showed that a physically salient distractor does not always capture attention or the eyes. Under certain top-down task sets, a salient distractor can be actively suppressed, avoiding capture. Even though previous studies showed that reaching movements are also influenced by salient distractors, it is unclear if and how a mechanism of active suppression of distractors would affect reaching movements. Active suppression might also explain why some studies find reaching movements to curve towards a distractor, while others find reaching movements to curve away. In this study, we varied the top-down task set in two separate experiments by manipulating the certainty about the target location. Participants had to reach for a diamond present among three circles. In Experiments 1 and 3, participants had to search for the reach targets; hence, the target’s location certainty was low. In Experiments 2 and 3, the target’s location was cued before the reach; hence, the target’s location certainty was high. We found that reaches curved towards the physically salient, color singleton, distractor in the search-to-reach task (Experiments 1 and 3), but not in the cued reach task (Experiments 2 and 3). Thus, the saliency of the distractor only attracted reaching movements when the certainty of the target’s location was low. Our findings suggest that the attractiveness of physically salient distractors to reaching movements depends on the top-down task set. The results can be explained by the effect of active attentional suppression on the competition between movement plans.

In everyday life, we manually interact with plenty of objects (e.g., by reaching towards a target object). The path of the reaching movement can be influenced by nontarget objects in the environment. If you need to quickly pick up your USB stick from a cluttered desk, you first have to search for the USB stick and then reach towards it to grab it. Would the reach path be influenced by other objects on the desk? And would this depend on their physical salience? Would the reach path be different if you do not have to search before reaching (e.g., if another person points towards the location of the USB stick)?

Physically salient distractors can capture visual attention and eye movements. For example, a salient distractor captures attention during a visual search task leading to slower response times (Theeuwes, 1992). Moreover, saccades are directed more often towards a high physically salient (HPS) distractor during search (Godijn & Theeuwes, 2002; McPeek, 2006; Theeuwes, Kramer, Hahn, Irwin, & Zelinsky, 1999). The presence of a distractor also influences the trajectory of saccades directed towards a visual target. Short latency saccades tend to curve towards a distractor, while long latency saccades tend to curve away from a distractor (McSorley, Haggard, & Walker, 2006; see also van Zoest, Donk, & Van der Stigchel, 2012). However, it has been shown that an active suppression process can prevent a physically salient distractor from capturing attention and eye movements (Lamy & Egeth, 2003; Lamy, Leber, & Egeth, 2004; Theeuwes & Burger, 1998). For example, cueing the target before the onset of a search display can omit attentional capture by a distractor (Yantis & Jonides, 1990). Also, distractors did not capture attention or eye movements in blocks with a relatively high proportion of distractor trials (Geyer, Müller, & Krummenacher, 2008). The active suppression of the distractor can take place shortly after the distractor is presented, but only under the right top-down task set. Task properties such as time of cueing, expectancies, relevancy, and feature overlap between the target and distractor, and many more, define the top-down task set. An important component of the top-down task set is the level of certainty of the distractor not being a target, which is again influenced by the level of certainty about the target location. For example, when the target location is validly cued on each trial before the target and distractor is presented, there is high certainty that the stimulus presented at the cued location is the target and, importantly, that stimuli presented at noncued locations are distractors. Moreover, the higher the feature overlap between target and distractor, the lower the certainty the distractor is not a target. Only when the certainty the distractor is not a target is high at the moment the distractor is presented, the distractor can be suppressed before it captures attention or the eyes. It has been argued that distractors produce an automatic attend-to-me signal that, under the right top-down task set, can be actively suppressed to prevent actual capture (Sawaki & Luck, 2010). It is unknown if the mechanism of fast active suppression also takes place in the planning of reaching movements.

Several studies found that reach trajectories are influenced by the presence of a salient distractor (Howard & Tipper, 1997; Kerzel & Schönhammer, 2013; Tipper, Howard, & Jackson, 1997; Welsh & Elliott, 2004; Welsh, Elliott, & Weeks, 1999; Wood et al., 2011). The influence of distractors on reach trajectories is often explained by competition between multiple movement plans (e.g., one towards the distractor and one towards the target; Cisek & Kalaska, 2005; McSorley, Haggard, & Walker, 2004; Tipper, Howard, & Houghton, 1998, 2000; see also Cisek & Kalaska, 2010; Gallivan, Barton, Chapman, Wolpert, & Flanagan, 2015; Gallivan, Chapman, Wolpert, & Flanagan, 2018; Herwig, 2015; Schneider, Einhauser, & Horstmann, 2013; Song, 2017). When competition between the two motor plans has not been resolved at the moment the reaching movement is executed, the initial direction of the movement will be diverted towards the distractor. When the competing movement plan towards the distractor is fully inhibited at the moment the reaching movement is executed, the movement’s initial direction will rather be away from the distractor. It has been argued that several experimental factors such as task instructions, the action-relevance of the distractor, the timing between target and distractor, or the cueing or priming procedures can influence the dynamics of the competition between target and distractor during movement planning and execution, and, hence, influence the movement trajectory (Song & Nakayama 2009). Experimental factors can also change the top-down task set under which a reaching movement is planned and performed. One important component of the top-down task set is the level of certainty about the target location. From the literature it seems that reaching movements to a target of which the location is certain tend to curve away from a distractor (e.g., Nissens & Fiehler, 2018; Moehler & Fiehler, 2017), whereas reaching movements tend to curve towards distractors when the target location is uncertain (e.g., Kerzel & Schönhammer, 2013; Moher, Anderson, & Song, 2015; Moher & Song, 2013; Neyedli & Welsh, 2012; Song & Nakayama, 2006, 2008; Welsh, 2011; Welsh & Elliott, 2004; Welsh et al., 1999; Wood et al., 2011). However, these studies differ in many design and task features; the top-down task set (i.e., level of target certainty) is only one. Hence, it is unclear whether the top-down task set can elucidate the mechanism determining when reaching movements curve away versus towards distractors.

In the present study, we examined whether and how the top-down task set (i.e., the certainty of the target location) influences the level and direction in which reaching curvature is affected by distractor saliency. We theorize that, with low target certainty and little active top-down control, the presentation of a salient distractor will activate a movement plan to the distractor, which will compete with the movement plan to the target. This will cause the reaching movement to curve towards the distractor. However, with high target certainty and active top-down control, the movement plan to the salient distractor will be suppressed shortly after its presentation. This will cause the reaching movement to curve away from the distractor. We hypothesize that reaches curve towards a distractor when there is uncertainty about the target location (e.g., when the target has to be searched; Moher et al., 2015; Moher & Song, 2013; Song & Nakayama, 2006, 2008). Curvature away from the distractor is expected when the target location is known (e.g., when the target is cued before the start of the reach; Nissens & Fiehler, 2018). In the first experiment, we asked participants to search for and reach towards a diamond shape presented among three circles. All shapes were presented either in the same color or one circle was presented in a low physically salient (LPS) or in a high physically salient (HPS) color. In the second experiment, the design was exactly the same, with one key difference: A cue was presented together with the onset of the shapes. Hence, participants did not have to search for the target, but could simply follow the cue and reach towards the indicated location. Based on our hypothesis, we expected that reaches would curve more toward the HPS distractor compared with the LPS distractor in the search-to-reach task (Experiments 1 and 3). In contrast, reaches should curve away from the HPS distractor compared with the LPS distractor in the cued reach task (Experiments 2 and 3).

## Experiment 1: Search-to-reach task

Experiment 1 aimed to investigate the effects LPS and HPS distractors have on reaching movements if participants need to search for the target in a stimulus display.

### Materials and methods

#### Participants

Twenty volunteers with reported normal or corrected-to-normal vision participated in the experiment. Two participants were excluded due to less than 50% of the trials meeting the inclusion criteria (see below) in at least one of the conditions of interest, resulting in a final sample of 18 participants (13 females, mean age 23 years). All participants were right-handed according to the Edinburgh Handedness Inventory (*M* = 85.4, *SD* = 19.9; Oldfield, 1971). All participants performed an Ishihara test (Ishihara, 2004) to ensure normal color vision. Participants gave written informed consent prior to the experiment and received course credits or financial compensation. The study was approved by the ethics committee of the Justus Liebig University Giessen, Department of Psychology and Sports Science, and was in accordance with the 2008 Declaration of Helsinki.

#### Experimental setup

Stimuli were created using Psychophysics Toolbox (Kleiner et al., 2007) in MATLAB (The MathWorks, Natick, MA, USA) and presented on a VPixx VIEWPixx monitor (1,920 × 1,200 pixels, 120 Hz; VPixx Technologies Inc., Saint-Bruno, QC, Canada). To enable the conversion of LAB color space to RGB color space, the monitor was color calibrated using a Konica Minolta Spectroradiometer CS-2000 (Konica Minolta Holdings Inc., Marunouchi, Tokyo, Japan). Reach movements were recorded with an optoelectronic motion tracking system (Optotrak Certus, Northern Digital Inc., Waterloo, ON, Canada), which registered an infrared marker placed on the fingernail of the right index finger with a sampling rate of 250 Hz. The motion tracking system was controlled via MATLAB using the Optotrak Toolbox created by V. H. Franz (http://www.ecogsci.cs.uni-tuebingen.de/OptotrakToolbox). Monocular movements of participants’ right eye were recorded via a head mounted video-based EyeLink II (SR Research, Mississauga, ON, Canada) with a sampling rate of 250Hz. Participants’ head was positioned on a chin rest at a distance of 48 cm from the screen.

#### Stimuli

**Fig. 1:**
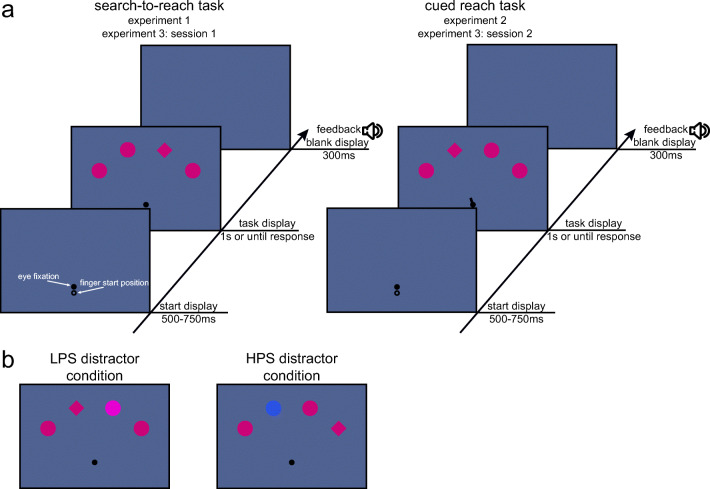
Experimental procedure. **a** Sequence of trial events. Participants had to reach for a diamond shape presented among circles. In Experiment 1 and in the first session of Experiment 3, the diamond shape had to be searched and reached for. In Experiment 2 and in the second session of Experiment 3, the diamond shape was cued and had to be reached for. **b** Example task display for the low physical salience (LPS) distractor condition (left) and high physical salience (HPS) distractor condition (right). In Experiments 1 and 2, both LPS and HPS distractors were presented. In Experiment 3, only the HPS distractor was presented

The experimental stimuli are depicted in Fig. 1a. The start display consisted of an eye fixation circle (0.42 vd = visual degrees radius) presented 2.5 vd below screen center. The outlined circle indicating the finger start position (0.42 vd radius) was presented 1.5 vd below the eye fixation circle. In the task display, the finger start position circle was removed and the four shapes (1.25 vd, 11mm radius), comprising three distractor circles and one target diamond, were positioned on an imaginary arc (10 vd, 88 mm radius) around eye fixation with 36 angular degrees between neighboring shapes.

All stimulus colors were defined in LAB color space which was created by the International Commission on Illumination (CIE) in an attempt to develop a perceptually uniform color space. In this color space, the distance between two colors in color space approximates the perceptual distance between those colors. LAB colors are defined in three coordinates: Lightness (≈luminance), A (green–red axis), and B (blue–yellow axis). The background (LAB: 50, 0, 0) and the shapes were isoluminant (i.e., same lightness). Eye fixation and finger start positions were in black (LAB: 0, 0, 0). The target color was red (LAB: 50, 75, 30); the low physical salience (LPS) color was reddish pink (LAB: 50, 91, 14); the high physical salience (HPS) color was blue (LAB: 50, 30, −75). The difference in chroma between the different shape colors was minimal. The distance in color space between the target color and the HPS color (114.24) was about five times the distance between the target color and the LPS color (22.63).

#### Procedure

The trial schedule is illustrated in Fig. 1a. The task of the participant was to reach as quickly and as accurately as possible for the diamond target shape while ignoring all other shapes and keeping their gaze on the eye fixation circle. The target appeared at any of the four shape locations with equal probability. On 50% of trials, all shapes had the same color (baseline condition). On 50% of trials one of the distractor circles was in a different color; of those trials, 50% were in the LPS color and 50% in the HPS color. The physical salient distractor circle could appear at any of the nontarget positions with equal probability.

The experiment consisted of two sessions, each consisting of 816 experiment trials, leading to a total of 1,632 trials per participant. Each session took about 60 to 90 minutes, including the setup of the participant and were performed on separate days. Participants took a break after each 102 trials. At the beginning of the first session, participants completed two practice blocks of 25 trials each. At the beginning of the second session, participants completed only one practice block of 25 trials. After each practice block the average reach latency was displayed and participants were encouraged to decrease reach latency. In the practice blocks all trials were baseline condition trials (i.e., all shapes had the same color).

Each trial started with the presentation of the start display. Then, the participants gaze and finger position were evaluated. When the participant’s finger was at the start circle (finger position within 5 mm in the *x, y* dimension and within 3 mm in *z* dimension from the center of the start circle), drift correction was performed, followed by a reevaluation of the finger position to ensure the participant had not moved their finger during drift correction. If participants did not have their finger at the start position after 600 ms the text “Finger not at start” was displayed until they moved their finger to the start position. If the finger position reevaluation was negative, the text “Hand moved too early. Trial restarts . . .” was displayed, and the trial was restarted. The fixation/start screen was presented for a randomized minimum time of 500 ms or 750 ms, or until gaze and finger position were evaluated positively. Next the task display consisting of the four shapes comprising three distractor circles and one target diamond was presented. Participants had to search for the diamond and then reach as quickly and as accurately as possible to it. Participants were instructed to fixate the fixation circle during the trial. Trials in which participants moved their eyes were excluded offline (see below). The task display was presented for a maximum of 1,000 ms. However, when a reach end was detected earlier the task display was removed 150ms later. A reach end was detected when the finger velocity dropped below 20 mm/s after moving more than 40 mm from the start position within 1,000 ms after the onset of the task display. Note that the reach onset detection procedure differed between the online and off-line analysis. The trial was evaluated as correct if the reach endpoint was within 28 mm from the center of the target shape. In any other case the trial was evaluated as incorrect. Participants received feedback about their performance after the offset of the task display in the form of a beep (high pitch = correct, low pitch = incorrect). The intertrial interval was 300 ms.

#### Analyses

Finger position coordinates were rotated online so that *x* and *y* were aligned to the horizontal and vertical axis of the monitor screen and, consequently, *z* was the axis perpendicular to the monitor screen. Small sections of missing reach data, due to the temporarily blocked view of the marker on the fingernail by the Optotrak, were interpolated for each dimension separately using the interp1 function within MATLAB. In the off-line analysis, the starting point of a reach was defined as the first sample of four consecutive vector velocity readings greater than 25 mm/s where there was a total acceleration of 20 mm/s^2^ across the four points. The end point of a reach was defined as the point in time when the velocity dropped below 20 mm/s (see Chapman & Goodale, 2010). Saccades’ start point and end point were detected online using minimum velocity and acceleration criteria of 30 vd/s and 8,000 vd/s^2^, respectively.

Trials were excluded when at least one of the following criteria was reached: a saccade of >2.5 vd was detected; the reach end was more than 28 mm away from the target center; the reach start was more than 10 mm from the finger start circle; the maximum reach velocity was >5,000 mm/s; the reach latency was <200 ms or >600 ms. Over all criteria and all participants, 7.64% of trials were excluded.

To determine whether the reaches deviated towards or away from the physical salient distractor, we calculated an attraction score (see Moher et al., 2015). The attraction score denotes the distance at a certain point along the trajectory between the baseline condition and one of the physically salient distractor conditions relative to the physically salient distractor’s location, with positive value denoting deviation towards (i.e., attraction) and negative value denoting deviation away. In more detail, using only the *x* and *y* coordinates, the reach movements were rotated and shifted so that the *x* coordinate of the end and start point equaled zero and the *y* coordinate of the start point equaled zero. To normalize the reach movement, reaches were resampled to 101 samples equally spaced along the amplitude (*y* dimension) using the normalizeFDA function of functional data analysis (FDA) for the reach trajectories toolbox in MATLAB (see Gallivan & Chapman, 2014; Ramsay & Silverman, 2005). For each combination of target and distractor location and each distractor condition (baseline vs. LPS vs. HPS) separately, we calculated the average reach movement. Next, for each combination of target and distractor location, we subtracted the baseline reach from the LPS and HPS reach. For target and distractor location combinations where the distractor was to the left of the target, the baseline corrected reach *x* values were multiplied by −1. Hence, positive *x* values denote deviation towards the physical salient distractor and negative *x* values denote deviation away from the physical salient distractor. The resulting *x* values of the reach movement are the values of the attraction score.

To determine when during the reaching movement the distractor attracted the trajectory, we performed a cluster-based analysis (see Maris & Oostenveld, 2007; Moher et al., 2015). The *t* statistic for the distractor attraction score was calculated for each point along the normalized space, then the largest cluster of consecutive *t* values, for which *p* < .05 was detected, and the sum of the *t* values in that cluster were calculated. Next, the order of *t* values was randomly permuted 100,000 times, and the same cluster analysis was performed to get a distribution of possible cluster sizes. A kernel density estimation was used to estimate a probability density function (PDF) from the distribution of possible cluster sizes. If the observed cluster size was significant, with *p* < .05 under the estimated PDF, the portion of the reaching movement related to this cluster is reported to be affected by the distractor.

Reach curvature was calculated by averaging the attraction score values. Hence, a positive reach curvature denotes deviation towards the distractor and negative values deviation away.

### Results

#### Main results

**Fig. 2:**
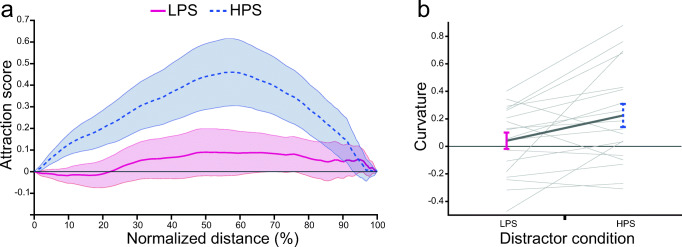
Results of Experiment 1: search-to-reach task. **a** Distractor attraction scores along the normalized movement amplitude. The blue line shows the attraction score for the high physical salience (HPS) distractor condition. The red line shows the attraction score for the low physical salience (LPS) distractor condition. Positive values indicate finger position deviated toward the distractor compared with baseline. **b** Curvature for the LPS distractor condition (red) and HPS distractor condition (blue). Positive values indicate that the reaching movement deviated toward the distractor. All error bars reflect between-subjects *SEM*

A one-way repeated-measures ANOVA with the factor distractor color (LPS vs. HPS vs. baseline) revealed that reaching movement curvature was influenced by the distractor color, *F*(2, 34) = 5.645, *p* = .008, η_p_^2^ = 0.249. Post hoc *t* tests revealed that reaching movement curvature was higher for the HPS distractor compared with the LPS distractor condition, *t*(17) = 2.706, *p*_*holm*_ = 0.045, *d*_*z*_ = 0.64. Furthermore, the movement curvature was significantly different from baseline in the HPS distractor condition, *t*(17) = 2.663, *p*_*holm*_ = 0.045, *d*_*z*_ = 0.63, but not in the LPS distractor condition, *t*(17) = 0.693, *p*_*holm*_ = 0.5, *d*_*z*_ = 0.16. Thus, reaches curved more towards the distractor presented in the HPS color compared with the LPS color (see Fig. 2b). This result was confirmed by the distractor attraction scores that were higher for the HPS than the LPS distractor condition from 6% to 69% of the reaching movement (see Fig. 2a). Hence, the reaching movement deviated stronger to the HPS distractor compared with the LPS distractor from 6% to 69% of the normalized reach amplitude.

The reach latency did not differ significantly between the distractor color conditions, *F*(2, 34) = 2.458, *p* = .101, η_p_^2^ = 0.126. Thus, the differences in reaching movement trajectory between the LPS, HPS, and baseline conditions are not related to differences in reach latency. However, the movement time was significantly different between the distractor color conditions, *F*(2, 34) = 8.699, *p* < .001, η_p_^2^ = 0.339. Post-hoc *t*-tests revealed that the movement time was longer when the HPS distractor was present (342 ms) compared to baseline (338 ms), *t*(17) = 4.361, *p*_*holm*_ < .001, *d*_*z*_ = 1.03. However, the movement time was not significantly different between LPS trials (339 ms) and HPS trials, *t*(17) = 2.421, *p*_*holm*_ = 0.054, *d*_*z*_ = 0.57, nor baseline, *t*(17) = 1.309, *p*_*holm*_ = 0.208, *d*_*z*_ = 0.31. The small increase in movement time on HPS trials compared with baseline trials is likely to be related to the increased curvature on HPS trials (see Table 1 for descriptive statistics).

**Table 1:** Descriptive statistics for reach movement data from Experiments 1–3

		Latency (ms)	Movement time (ms)
Experiment 1			
	Baseline	294 ± 24	338 ± 46
	LPS	295 ± 26	339 ± 45
	HPS	296 ± 27	342 ± 44
Experiment 2			
	Baseline	291 ± 48	318 ± 51
	LPS	291 ± 48	317 ± 50
	HPS	290 ± 49	319 ± 50
Experiment 3			
	Search baseline	301 ± 30	298 ± 32
	Search HPS	303 ± 31	304 ± 32
	Cue baseline	284 ± 86	301 ± 35
	Cue HPS	285 ± 36	301 ± 36

*Note.* Error terms reflect standard deviation. LPS = low physically salient; HPS = high physically salient

#### Exploratory results

**Fig. 3:**
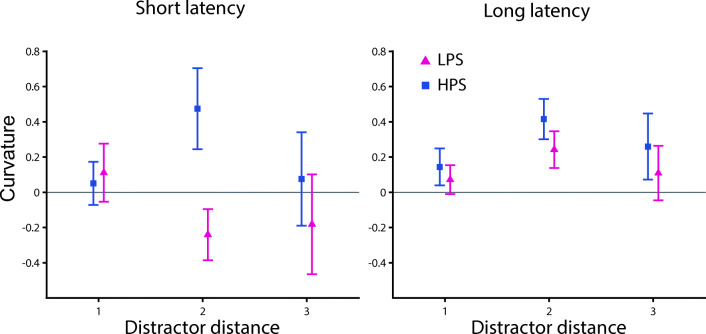
Exploratory results of Experiment 1: search-to-reach task. Curvature for the low physical salience (LPS) distractor (red) and high physical salience (HPS) distractor (blue) condition depending on the distance between the position of the target and the distractor for short latency (left) and long latency (right) reaching movements. All error bars reflect between-subjects *SEM*

From the eye movement literature, it is known that the effect of distractor presence on movement properties can depend on the latency of the movement (e.g., McSorley et al., 2006) or the distance between the target and the distractor (e.g., Godijn & Theeuwes, 2002). Similar effects have been found for reaching movements showing a stronger movement curvature for distractors located close than far from the target (Moehler & Fiehler, 2017). Moreover, shorter reach latencies seem to lead to more curved trajectories in a search-to-reach task (Song & Nakayama, 2008). Based on these previous findings, we performed exploratory analyses and examined whether reach latency and/or the distance between the target and the distractor interacted with the influence of the physical salience of the distractor on reach curvature (see Fig. 3). On the individual participant level, we performed a median-split based on reach latency (short vs. long) for each combination of target and distractor location, separately. Next, we grouped the combinations of target and distractor position based on the distance between them (one, two, or three). A three-way repeated-measures ANOVA was performed with the factors reach latency (short vs. long), distractor distance (one vs. two vs. three), and distractor color (LPS vs. HPS). The reported *p* values were Greenhouse–Geisser corrected for sphericity where applicable; noncorrected degrees of freedom are reported. Apart from the main effect of distractor color, *F*(1, 17) = 6.973, *p* = .017, η_p_^2^ = 0.291, a two-way and a three-way interaction effect were significant: distractor distance, and distractor color, *F*(2, 34) = 4.328, *p* = .021, η_p_^2^ = 0.203, and distractor distance, distractor color, and reach latency, *F*(2, 34) = 3.951, *p* = .029, η_p_^2^ = 0.189. The two-way interaction effect seems related to a larger difference in curvature between the LPS and HPS distractor when the distractor was two positions away from the target (see Fig. 3). Post hoc *t* tests revealed that the difference between the LPS and HPS distractor was only significant when the distractor was two positions away from the target, LPS vs. HPS for distractor distance one, *t*(17) = 0.169, *p*_*holm*_ = 0.868, *d*_*z*_ = 0.040; distance two: *t*(17) = 3.300, *p*_*holm*_ = 0.012, *d*_*z*_ = 0.778; distance three: *t*(17) = 1.037, *p*_*holm*_ = 0.628, *d*_*z*_ = 0.244. Moreover, the three-way interaction effect seems to be driven by a larger difference in curvature between the LPS and HPS distractor on short compared with long reach latency trials when the distractor was two positions away from the target (see Fig. 3). Post hoc *t* test revealed that the difference between LPS and HPS distractor was only significant on short latency saccades when the distractor was two positions away from the target, LPS vs. HPS for short latency and distractor distance one: *t*(17) = 0.461, *p*_*holm*_ = 1.000, *d*_*z*_ = 0.109; distance two: *t*(17) = 3.937, *p*_*holm*_ = 0.006, *d*_*z*_ = 0.928; distance three: *t*(17) = 0.746, *p*_*holm*_ = 1.000, *d*_*z*_ = 0.176; long latency and distractor distance one: *t*(17) = 0.405, *p*_*holm*_ = 1.000, *d*_*z*_ = 0.096; distance two: *t*(17) = 0.799, *p*_*holm*_ = 1.000, *d*_*z*_ = 0.188; distance three: *t*(17) = 0.649, *p*_*holm*_ = 1.000, *d*_*z*_ = 0.153. All other main and interaction effects were nonsignificant, distractor distance: *F*(2, 34) = 1.335, *p* = .277, η_p_^2^ = 0.073; reach latency: *F*(1, 17) = 1.364, *p* = .259, η_p_^2^ = 0.074; Distractor Distance × Reach Latency: *F*(2, 34) = 0.686, *p* = .466, η_p_^2^ = 0.039; Distractor Color × Reach Latency: *F*(2, 34) = 1.181, *p* = .292, η_p_^2^ = 0.065.

## Experiment 2: Cued reach task

Experiment 2 aimed to investigate the effect a low and high physically salient distractor have on reaching movements when the target is cued upon stimulus display presentation.

### Materials and methods

#### Participants

Twenty-two volunteers with reported normal or corrected-to-normal vision participated in the experiment. Four participants were excluded due to less than 50% of trials meeting the inclusion criteria in at least one of the conditions of interest, resulting in a final sample of 18 participants (10 females, mean age 24 years). All participants were right-handed according to the Edinburgh Handedness Inventory (*M* = 81, *SD* = 18.4; Oldfield, 1971). All participants performed an Ishihara test (Ishihara, 2004) to ensure normal color vision. Participants gave written informed consent prior to the experiment and received course credits or financial compensation. The study was approved ethics committee of the Justus Liebig University Giessen and was in accordance with the 2008 Declaration of Helsinki.

#### Experimental setup

The setup was the same as in the first experiment.

#### Stimuli

The stimuli were the same as in Experiment 1, except from one difference: To transform the search-to-reach task into a cued reach task, a black line (0.39 vd; LAB: 0, 0, 0) was added to the task display pointing out from the eye fixation circle towards the target location. Please note that the cue appeared simultaneously with the shapes. The cue was presented together with, and not before the onset of, the shapes to avoid preplanning of the reaching movement (i.e., to be sure the shapes were present when the movement was planned).

#### Procedure

The procedure was the same as in the first experiment, with the only difference that participants were now told to reach towards the shape indicated by the centrally presented cue (see Fig. 1a).

#### Analysis

The analysis was the same as in Experiment 1. We used the same exclusion criteria, resulting in an overall trial exclusion rate of 7.79%.

### Results

#### Main results

**Fig. 4:**
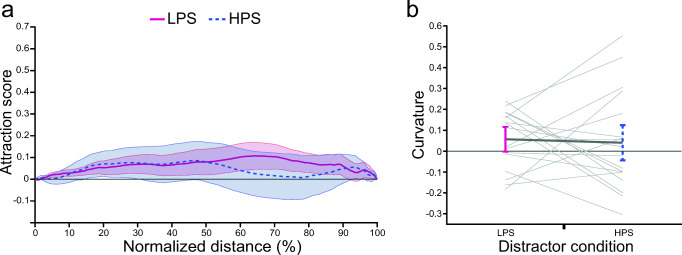
Results of Experiment 2: cued reach task. **a** Distractor attraction scores along the normalized movement amplitude. The blue line shows the attraction score for the high physical salience (HPS) distractor condition. The red line shows the attraction score for the low physical salience (LPS) distractor condition. Positive values indicate finger position deviated toward the distractor compared with baseline. **b** Curvature for the LPS distractor condition (red) and HPS distractor condition (blue). Positive values indicate that the reaching movement deviated towards the distractor. All error bars reflect between-subjects *SEM*

A one-way repeated-measures ANOVA with the factor distractor color (LPS vs. HPS vs. baseline) revealed that reaching movement curvature was not influenced by the distractor color, *F*(2, 34) = 0.642, *p* = .533, η_p_^2^ = 0.036. Also, the attraction score was not significantly different between any of the conditions along the normalized reach amplitude (see Fig. 4a).

The reach latency did not differ significantly between the distractor color conditions, *F*(2, 34) = 0.527, *p* = .595, η_p_^2^ = 0.030. The movement time was significantly different between the distractor color conditions, *F*(2, 34) = 4.241, *p* = .023, η_p_^2^ = 0.20. However, none of the post hoc *t* tests were significant after Holm correction, HPS vs. LPS: *t*(17) = 2.530, *p*_*holm*_ = 0.066, *d*_*z*_ = 0.596; HPS vs. Baseline: *t*(17) = 1.755, *p*_*holm*_ = 0.194, *d*_*z*_ = 0.414; LPS vs. Baseline: *t*(17) = 1.407, *p*_*holm*_ = 0.194, *d*_*z*_ = 0.332 (see Table 1 for descriptive statistics).

#### Exploratory results

**Fig. 5:**
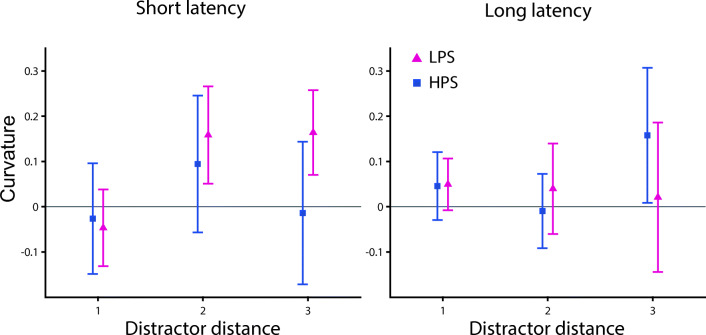
Exploratory results Experiment 2: cued reach task. Curvature for the low physical salience (LPS) distractor (red) and high physical salience (HPS) distractor (blue) condition depending on the distance between the position of the target and the distractor for short latency (left) and long latency (right) reaching movements. All error bars reflect between-subjects *SEM*

As in Experiment 1, we tested whether the effect of distractor saliency on reach curvature depends on the reach latency and the distance between the target and the distractor (see Fig. 5). We performed a three-way ANOVA with the factors reach latency (short vs. long), distractor distance (one vs. two vs. three), and distractor color (LPS vs. HPS). The interaction effect between reach latency and distractor color was not significant, *F*(1, 17) = 0.986, *p* = .335, η_p_^2^ = 0.055. None of the main or other interaction effects were significant, distractor distance: *F*(2, 34) = 0.123, *p* = .720, η_p_^2^ = 0.018; reach latency: *F*(1, 17) = 0.005, *p* = .943, η_p_^2^ = 0.000; distractor color: *F*(1, 17) = 0.137, *p* = .716, η_p_^2^ = 0.008; Distractor Distance × Reach Latency: *F*(2, 34) = 0.544, *p* = .528, η_p_^2^ = 0.031; Distractor Distance × Distractor Color: *F*(2, 34) = 0.150, *p* = .783, η_p_^2^ = 0.009; Distractor Distance × Distractor Color × Reach Latency: *F*(2, 34) = 0.745, *p* = .434, η_p_^2^ = 0.042. Thus, the influence of distractor salience does not significantly depend on the reach latency.

## Experiment 3: Cued reach versus search-to-reach tasks

Experiment 3 aimed to directly compare the effect of a highly physically salient distractor on reaching movements in a search-to-reach task and a cued reach task by conducting a within-subjects experiment.

### Materials and methods

#### Participants

Thirty-nine volunteers with reported normal or corrected-to-normal vision participated in the experiment. Eleven participants were excluded due to less than 50% of trials meeting the inclusion criteria in at least one of the conditions of interest, resulting in a final sample of 28 participants (21 females, mean age 25 years). The sample size was estimated using G*Power (Faul, Erdfelder, Lang, & Buchner, 2007) based on the effect size of the between subject effect of experimental task (Experiment 1 vs. Experiment 2) on reach curvature during HPS trials, *d*_*z*_ = 0.609, with *α* error probability = 0.05, and power = 0.85. This resulted in a sample size estimate of 27 participants. Twenty-eight participants were tested to counterbalance the order of the task between participants. All participants were right-handed according to the Edinburgh Handedness Inventory (*M* = 82, *SD* = 16.3; Oldfield, 1971). All participants performed an Ishihara test (Ishihara, 2004) to ensure normal color vision. Participants gave written informed consent prior to the experiment and received course credits or financial compensation. The study was approved by the ethics committee of the Justus Liebig University Giessen and was in accordance with the 2008 Declaration of Helsinki.

#### Experimental setup

The setup was the same as in Experiments 1 and 2.

#### Stimuli

The stimuli were the same as in Experiments 1 and 2. However, only the baseline and HPS distractor conditions were included.

#### Procedure

The procedure was largely the same as in Experiments 1 and 2, with some important differences (see Fig. 1a): (i) Participants performed two sessions on two separate days. In one session they performed the cued reach task and on the other the search-to-reach task. The order was counterbalanced between participants. (ii) During the practice blocks, participants received written feedback about their performance presented at fixation level for 750 ms on every trial (correct, too slow, eyes moved, wrong shape, or correct but too slow). (iii) In one third of the trials, the HPS distractor was presented, in the other two thirds of trials, no salient distractor was presented (i.e., baseline trials). Each session consisted of 720 trials divided into six blocks. (iv) Participants were encouraged to perform their reach as quickly as possible by introducing a variable deadline of movement duration. If participants reached the target after this deadline the trial would be marked as too slow and they would receive negative feedback. The variable deadline was based on the 80th percentile of the time it took to reach the target for every participant and target location separately. Based on the average performance in Experiments 1 and 2, the variable deadline was set to 650 ms until participants performed 10 correct trials to each target location.

#### Analysis

The analysis was the same as in Experiments 1 and 2. We used the same exclusion criteria resulting in an overall trial exclusion rate of 2.45%.

### Results

#### Main results

**Fig. 6:**
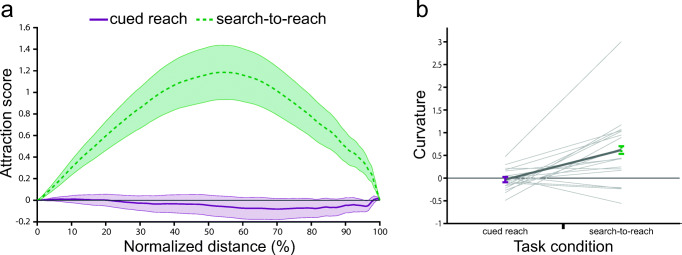
Results of Experiment 3. **a** Distractor attraction scores along the normalized movement amplitude. The green line shows the attraction score for the high physical salience (HPS) distractor in the search-to-reach task. The purple line shows the attraction score for the HPS distractor in the cued reach task. Positive values indicate that the finger position deviated toward the distractor compared with baseline. **b** Curvature for the cued reach task (purple) and search-to-reach task (green). Positive values indicate that the reaching movement deviated towards the HPS distractor. All error bars reflect between-subjects *SEM*

The two-way ANOVA with factors distractor color (baseline vs. cue) and task (search-to-reach vs. cued reach) revealed a significant main effect of distractor color, *F*(1, 27) = 12.91, *p* = .001, η_p_^2^ = 0.323, and task, *F*(1, 27) = 12.86, *p* = .001, η_p_^2^ = 0.323, and a significant interaction effect between distractor color and task, *F*(1, 27) = 24.89, *p* < .001, η_p_^2^ = 0.48. Post hoc *t* tests revealed that reaching curvature towards the HPS distractor was larger during the search-to-reach task compared with the cued reach task (see Fig. 6b), *t*(27) = 4.989, *p*_*holm*_ < .001, *d*_*z*_ = 0.943. The curvature on HPS trials compared to baseline was larger in the search-to-reach task, *t*(27) = 4.497, *p*_*holm*_ < .001, *d*_*z*_ = 0.850, but not different in the cued reach task, *t*(27) = 0.612, *p*_*holm*_ = 0.546, *d*_*z*_ = 0.116. The attraction score (see Fig. 6a) revealed that in the search-to-reach task the reaching movement was attracted towards the HPS distractor from 3% to 97% along the normalized amplitude compared with the cued reach task and from 3% to 99% compared with baseline.

To investigate whether the reach latency differed between the two tasks and the distractor conditions, a two-way ANOVA with factors task (search vs. cue) and distractor condition (HPS vs. baseline) was performed which revealed a main effect of task, *F*(1, 27) = 8.652, *p* = .007, η_p_^2^ = 0.243, and a main effect of distractor condition, *F*(1, 27) = 6.088, *p* = .020, η_p_^2^ = 0.184. The interaction effect was not significant, *F*(1, 27) = 0.339, *p* = .565, η_p_^2^ = 0.012. Post hoc *t* tests revealed that the latency was higher in the search-to-reach task (302 ms) compared with the cued reach task, 285 ms, *t*(27) = 4.183, *p*_*holm*_ < .001, *d*_*z*_ = 0.791. Reaching movements were initiated later when an HPS distractor was present (294 ms) compared with baseline, 293 ms, *t*(27) = 2.798, *p*_*holm*_ = .007, *d*_*z*_ = 0.529.

Moreover, a two-way ANOVA was performed to investigate whether the movement time differed between task (search vs. cue) and distractor conditions (HPS vs. baseline). A main effect of distractor condition, *F*(1, 27) = 18.120, *p* < .001, η_p_^2^ = 0.402, and interaction between task and distractor condition, *F*(1, 27) = 20.015, *p* < .001, η_p_^2^ = 0.426, was observed. However, there was no main effect of task, *F*(1, 27) = 0.0001, *p* = .98, η_p_^2^ = 0.000. Post hoc *t* tests revealed that the movement duration was longer on HPS (294 ms) compared to baseline, 293 ms trials, *t*(27) = 4.001, *p*_*holm*_ < .001, *d*_*z*_ = 0.756. Regarding the interaction effect, the difference between HPS and baseline condition was significant for the search-to-reach task, *t*(27) = 5.03, *p*_*holm*_ < 0.001, *d*_*z*_ = 0.951, with longer movement times when the HPS distractor was present (304 ms) compared with baseline (298 ms); but not for the cued reach task, *t*(27) = 0.407, *p*_*holm*_ = 0.687, *d*_*z*_ = 0.077.

#### Exploratory results

**Fig. 7:**
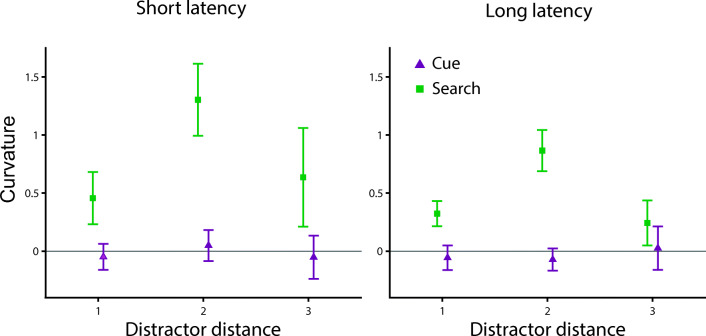
Exploratory results of Experiment 3. Curvature for the cued reach task (purple) and search-to-reach task (green) over the distance between the position of the target and the distractor for short latency (left) and long latency (right) reaching movements. All error bars reflect between-subjects *SEM*

Similarly, as in Experiments 1 and 2, we tested whether the effect of saliency on reaching movements depended on the distance between the target and distractor, the latency of the movement or any interaction of those factors with the effect of task (see Fig. 7). Therefore, we performed a three-way ANOVA with factors reach latency (short vs. long), distractor distance (one vs. two vs. three), and task (cue vs. search). We found a main effect of task, *F*(1, 27) = 16.969, *p* < .001, η_p_^2^ = 0.386, and distractor distance, *F*(2, 54) = 4.960, *p* = .013, η_p_^2^ = 0.155; but no main effect of latency, *F*(1, 27) = 1.472, *p* = .24, η_p_^2^ = 0.052. Furthermore, the interaction effect between task and distractor distance was trending, *F*(2, 54) = 3.138, *p* = .068, η_p_^2^ = 0.104. All other interaction effects were nonsignificant, Task × Reach Latency: *F*(1,27) = 1.919, *p* = .177, η_p_^2^ = 0.066; Distractor Distance × Reach Latency: *F*(2,54) = 0.347, *p* = .682, η_p_^2^ = 0.013; Task × Distractor Distance × Reach Latency: *F*(2,54) = 0.324, *p* = .705, η_p_^2^ = 0.012. The post hoc *t* tests for the effect of distractor distance revealed a difference between distractor distance two and one (0.54 vs. 0.17), *t*(27) = 3.399, *p*_*holm*_ = 0.003, *d*_*z*_ = 0.642, and distractor distance two and three (0.54 vs. 0.21), *t*(27) = 2.413, *p*_*holm*_ = 0.035, *d*_*z*_ = 0.456, but not between distractor distance one and three (0.17 vs. 0.21), *t*(27) = 0.313, *p*_*holm*_ = 0.757, *d*_*z*_ = 0.059. Hence, the HPS distractor attracted the reaching movements more when the distractor was two positions away from the target. This is similar to the finding in Experiment 1, where we observed a larger difference in curvature between the LPS and HPS condition when the distractor was two positions away from the target.

## General discussion

The present study investigated how the top-down task set (i.e., the certainty of the target location) influences the direction and magnitude of the effect that a physically salient distractor has on reaching trajectories. In the search-to-reach task (Experiment 1), when certainty about the target location was low, reaches curved more towards the high physically salient (HPS) distractor than to the low physically salient (LPS) distractor relative to when there was no physically salient distractor present (baseline). Moreover, the trajectory deviated more towards the HPS distractor compared with the LPS distractor from 6% to 69% of the reaching movement. However, in the cued reach task (Experiment 2), when certainty about the target location was high, there was no difference in reach trajectory when there was an HPS distractor or an LPS distractor present compared with baseline. In a direct comparison (Experiment 3), the differences between the search-to-reach task and cued reach task were confirmed. Hence, we showed that a physically salient distractor attracts reach trajectories when the certainty of the target location is low, but not when the certainty of the target location is high.

Our findings suggest that the effect of distractors on reaching movement trajectories is influenced by the level of certainty of the target location during movement planning. In line with previous studies (Moher et al., 2015; Moher & Song, 2013; Song & Nakayama, 2006, 2008; Welsh, 2011; Welsh & Elliott, 2004; Welsh et al., 1999) and with the first part of our hypothesis, we found attraction towards the distractor when certainty was low (Experiments 1 and 3, search-to-reach task). In Welsh et al. (1999), for example, participants had to reach towards a red target that was presented at a random location on a 3 × 3 grid. Note that the certainty of the target location was low. On some trials a yellow distractor was presented at another location on the grid. They found that the reaching movements curved towards the distractor. Our results add to the evidence by showing that reaching trajectories are attracted to an HPS distractor in a search-to-reach task. The results from Experiment 1 can be explained by competition between multiple movement plans (Cisek & Kalaska, 2005; McSorley et al., 2004; Tipper et al., 1998, 2000; see also Cisek & Kalaska, 2010; Gallivan et al., 2015; Gallivan et al., 2018; Herwig, 2015; Schneider et al., 2013; Song, 2017). During movement preparation, a movement plan towards the HPS distractor is activated along with a movement plan towards the target. When a reaching movement is executed, the activation of the movement plan towards the distractor is not fully inhibited, which leads to a reaching movement in which the initial direction is shifted towards the distractor location.

We did not corroborate the second part of our hypothesis, as we did not observe deviation away from the distractor when certainty of the target’s location was high (Experiments 2 and 3, cued reach task). We expected that the cue would lead to an active inhibition of the movement plan towards the HPS distractor that would cause the reaching movement to curve away from the distractor. However, this is not what we observed. Based on our results, it is more likely that the suppression occurs on the level of visual attention. The HPS distractor might trigger an “attend-to-me” signal, which, given the top-down task set of high target certainty, leads to active suppression of the distractor (Sawaki & Luck, 2010). We argue that the attentional map, with an already suppressed distractor, is then transferred to the motor map. There, the movement plan to the distractor is not activated and does not lead to competition with the movement plan. Hence, no curvature away or towards the distractor is observed. However, alternative explanations are warranted: (i) The cue could have activated the movement plan towards the target in a strong fashion relative to the saliency driven activation of the movement plan towards the distractor. However, during the cued reach task, the cue driven movement plan activation might be stronger than the saliency driven movement plan activation, and therefore the movement plan towards the distractor is too weak to affect competition. Consequently, no influence of the distractor on curvature is observed. (ii) Possibly, participants learned to produce a motor command from memory following the cue without engaging in visually guided motor planning. Hence, no influence of the distractor on curvature is observed. We want to point out that all three explanations (distractor suppression, strong target activation, and motor command from memory) predict the movement latency to be shorter in the cued reach task compared with the search-to-reach task, as we did observe. All explanations assume less or no competition for movement selection, which would assume a shorter duration of target selection.

In the study by Welsh and Elliott (2005) a cue presented 1–1.5 seconds prior to target/distractor onset was valid in 75% of trials. On half of the invalid cue trials the distractor was presented at the precued location. They found that the reach was influenced by the distractor when the distractor was at the precued location, but not when the target was validly precued. They argue that the precue leads to a preactivation of the movement plan towards the cued location during movement selection. Therefore, when the target is validly cued, the movement plan to the distractor is activated too late or too weak to influence competition. When the distractor is cued, the activation of the movement plan towards the distractor is increased and does compete with the movement plan towards the target. Similarly, as in our study, the distractor did not influence reaching when the target was cued. However, in their study the cue was presented 1–1.5 seconds before target/distractor was presented. Already before target presentation, a movement plan towards the cued location could have been activated. This was not possible in our study since the cue was presented together with the onset of the target and distractor shapes. Even though our results can be explained by a strong activation of the movement plan towards the target, this could not have been in a preplanned fashion. Alternatively, by cueing the target, the salient distractor could have been suppressed on the attentional priority map which then also affects the motor map. As a consequence, the movement plan towards the distractor would not be activated and thus would not compete with the movement plan towards the target.

Our findings show similarities with the findings in Yantis and Jonides (1990). In their second experiment (reaction time task), a valid cue was either presented before, together, or shortly after the onset of the search display. They showed that an onset distractor slowed down response times when the cue was presented together with or after the search display, but not when the cue was presented earlier. Hence, they show that whether a distractor captures attention depends on the level of certainty about the target location. It has been argued that distractors produce an automatic attend-to-me signal that can be actively suppressed in a top-down fashion to prevent actual capture of attention (Sawaki & Luck, 2010). Whether the distractor is actively suppressed, avoiding capture, depends on the top-down control settings which are task-dependent (Geyer et al., 2008; Lamy & Egeth, 2003; Lamy et al., 2004; Theeuwes & Burger, 1998). An important factor is the certainty of where the target is or, in other words, the certainty that the distractor location is different from the target location. If the certainty of the target location is high the attend-me-signal triggered by the distractor is more likely to be followed by an active suppression of the distractor to avoid attentional capture than when the certainty of the target location is low. The present study shows the consequence of active attentional suppression on the planning of reaching movements. The presentation of the HPS distractor leads to an attend-to-me signal. In the search-to-reach task, this leads to attentional capture and an attraction of the reaching movement to the HPS distractor. However, in the cued reach task, the attend-to-me signal, given the high target certainty, is followed by an active attentional suppression of the HPS distractor. The attentional suppression prevents the attraction of the reaching movements to the HPS distractor.

Our results show that the top-down task set influences the effect a salient distractor has on reaching trajectories. A suggestion for future research to delve into task properties is to vary the level of certainty of the distractor location This suggestion taps into the idea of anticipatory distractor suppression, which has been shown in visual search (e.g., Wang, van Driel, Ort, & Theeuwes, 2019). In case the anticipatory distractor suppression would transfer to reaching movements, we would expect reaching movements to curve less towards distractors presented at an expected location during a search-to-reach task.

In light of a recent paper (Hommel et al., 2019) phrasing valid concerns about the use of the term “attention” and the confusion that comes with it, we would like to clarify how we define attention. In general, throughout this paper, attention refers to the processes influencing the selection of stimuli or locations within the visual domain, which is seen as the consequence of activation and competition in a visual priority map. Correlates of visual priority maps have been found in the intermediate layers of the superior colliculus, the intraparietal cortex and the frontal eye fields (Bisley & Goldberg, 2010; Thompson & Bichot, 2005; White et al., 2017). Moreover, attentional capture and suppression is seen as a consequence of, respectively, the prioritization and inhibition of, for example, a stimulus feature or spatial location within the visual priority map. Priority signals can come from different sources that are assumed to be initiated in different brain regions. Stimulus salience might originate from early visual cortex and saliency maps in the superficial layers of the superior colliculus (Itti & Koch, 2001). The neural origin of top-down influences is not well understood and might not necessarily be traced back to one neural substrate. However, some evidence has suggested that the anterior cingulate and orbitofrontal cortex play a role in top-down instruction-related value sets (Kennerley, Behrens, & Wallis, 2011).

In this study we show that task-dependent factors do influence the effect a physically salient distractor has on reaching movements. We argue that the top-down task set determines whether the distractor captures visual attention or not, which in turn defines the initial level of activation of movement plans towards the target and distractor and the subsequent amount of competition between them.
